# Assessment of Genetic Diversity and Genetic Structure of *Saussurea medusa* (Asteraceae), a “Sky Island” Plant in the Qinghai–Tibet Plateau, Using SRAP Markers

**DOI:** 10.3390/plants12132463

**Published:** 2023-06-27

**Authors:** Jun Wang, Wei Dai, Jie Chen, Kunhao Ye, Qianglong Lai, Dan Zhao

**Affiliations:** Crop Characteristic Resources Creation and Utilization Key Laboratory of Sichuan Province, Mianyang Academy of Agricultural Sciences, Mianyang 621022, China; wangjunmyaas@outlook.com (J.W.); davidwxl17@foxmail.com (W.D.); chenjie_myaas@163.com (J.C.); ye_kunhao@outlook.com (K.Y.); lql930615@163.com (Q.L.)

**Keywords:** *Saussurea medusa*, sky island, genetic diversity, genetic structure, population differentiation, climatic factors

## Abstract

*Saussurea medusa* Maxim. is a typical “sky island” species and one with the highest altitude distributions among flowering plants. The present study aimed at analyzing the genetic diversity and population structure of 300 *S. medusa* accessions collected from 20 populations in the Qilian Mountains in the northeastern Qinghai–Tibet Plateau (QTP), using sequence-related amplified polymorphism (SRAP) markers. A total of 14 SRAP primer combinations were employed to analyze genetic diversity and population structure across all accessions. Out of 511 amplified bands, 496 (97.06%) were polymorphic. The populations in the eastern Qilian Mountains had significantly higher genetic diversity than those in the central and western groups. Population structure analysis revealed greater genetic differentiation among populations with a *Gst* of 0.4926. UPGMA-based clustering classified the 300 *S. medusa* accessions into 3 major clusters, while the Bayesian STRUCTURE analysis categorized them into 2 groups. Correlation analyses showed that the genetic affinity of the populations was based on differences in geographical distance, moisture conditions, and photothermal conditions between the habitats. This study represents the first comprehensive genetic assessment of *S. medusa* and provides important genetic baseline data for the conservation of the species.

## 1. Introduction

*S. medusa*, a rare monocarpic perennial herb belonging to the genus *Saussurea*, family Asteraceae, grows on rocky beaches, rock cracks, and gravel hillsides at a height of 3000–5600 m above sea level [1]. In China, it is mainly distributed throughout the Qinghai–Tibet Plateau (QTP) and its surrounding alpine areas, including the Qinghai, Gansu, Sichuan, and Yunnan provinces and the Tibet and Xinjiang autonomous regions [2]. In traditional Tibetan and Uygur folk medicine, *S. medusa* is widely used to treat rheumatoid arthritis and gynecological diseases. Modern pharmacological experiments have shown that *S. medusa* has anti-inflammatory, analgesic, anti-tumor, anti-oxidative, hypolipidemic, and uterine contraction stimulation effects [3,4,5,6,7]. In the past 2 decades, the focus on phytochemical research resulted in the identification of >70 chemical components in the whole plant, the main components being flavonoids, coumarins, phenylpropanoids, and lignans [8,9,10,11,12,13,14].

In addition, its populations are relatively isolated by steep valleys and high mountain ridges, showing the typical distribution model of “sky island” [15,16,17]. Limited gene flow due to habitat isolation could lead to stronger genetic differentiation among populations compared with plants found in less isolated habitats and/or lower altitudes [18]. However, populations that do not exchange genes with others decrease their genetic diversity, thus increasing their risk of extinction. *S. medusa* has a small range of distribution and population size, which could have produced its loss of alleles and genetic variability due to genetic drift. As one of the three main forms of biodiversity, genetic diversity provides raw materials for the evolution of species, thereby providing potential for adaptation to constantly changing environments [19]. The mating system has a significant impact on the maintenance of population genetic diversity and ultimately determines how variations are transmitted across generations [20]. *S. medusa* is a predominantly outcrossing plant, mainly pollinated by insects (Bombus spp., Apidae), despite a certain degree of self-pollination in the absence of pollinating agents [21]. However, long-term self-fertilization results in reduced fitness in the progeny and decreased genetic diversity in the species [22]. In addition, the genetic variation in quantitative traits can be influenced by environmental changes, although there is no evidence to suggest the universality of the reduction or increase in variation caused by environmental change [23,24]. Plant populations exhibit local adaptability to their respective habitats, as each population evolves toward its own optimum through specific genetic selection [24]. Many studies have confirmed the correlation between genetic variation and environmental factors, especially climate factors such as light regime, precipitation, and temperature [25,26,27,28]. So far, the genetic background of *S. medusa*, particularly in terms of genetic diversity and genetic structure, has been rarely studied owing to its small population size and sampling difficulties.

Currently, molecular genetic markers are a reliable method for the population-level genetic analysis of alpine plants. Gaudeul et al. [29] used an amplified fragment length polymorphism (AFLP) marker to measure the genetic diversity of 14 *Eryngium alpinum* populations (327 individuals) in the European Alps. Pluess et al. [30] used a random amplified polymorphic DNA (RAPD) marker to study the genetic diversity within and among 20 populations of *Geum reptans*, an outcrossing clonal plant species in the Swiss Alps. Sequence-related amplified polymorphism (SRAP) is an effective and convenient molecular marker for assessing biological genetic diversity based on PCR methods. This technology presents several advantages over other marker technologies, including its high throughput rates, high commonality, easy band separation and sequencing, and its targeting of open reading frames (ORFs) [31,32,33,34]. SRAP markers have been widely used in the genetic diversity analysis of various genera of Asteraceae, including *Cynara*, which is a close relative of *Saussurea* [35,36,37]. However, there was no record of its application to *Saussurea* in our literature search.

In this study, we used 14 pairs of SRAP markers to evaluate the genetic diversity and population structures of 300 *S. medusa* accessions collected from the Qilian Mountains in the northeastern QTP. We also analyzed the objective factors that may contribute to population differentiation, considering geographical distance and climatic factors. To our knowledge, this is the first report on the genetic diversity and population structure of *S. medusa*.

## 2. Results

### 2.1. Polymorphism Analysis of SRAP Amplified Products

A total of 88 pairs of SRAP primer combinations were preliminarily screened. The amplification bands of 14 pairs were clear and evenly distributed, which were used to estimate the genetic diversity of *S. medusa*. The primer sequences are shown in Table 1. These SRAP primer combinations amplified 511 loci (496 polymorphic loci, 97.06%) with a size range of 45–1600 bp and quantity range of 26–44 per primer. The primer combination ME8/EM9 showed the lowest polymorphism (88.46%), while the highest polymorphism (100%) was detected by the primer combinations ME1/EM4, ME3/EM1, ME3/EM3, ME4/EM3, ME4/EM4, and ME4/EM5. The range of polymorphism information content (*PIC*) was from 0.48 to 0.50. Examples of the PCR amplification results with various primer combinations are shown in Figure S1.

### 2.2. Genetic Diversity and Genetic Structure Analyses

Table 2 shows the results obtained using the POPGENE software for the genetic diversity parameters. The *Na*, *Ne*, *He*, and *I* values at the species level were 1.9706, 1.4598, 0.2757, and 0.4237, respectively. At the population level, assuming a Hardy–Weinberg equilibrium, *pop* SJC (*Na* = 1.5558, *Ne* = 1.3279, *He* = 0.1913, *I* = 0.2865) had the highest genetic diversity, followed by *pop* SNK, whereas *pop* LMX (*Na* = 1.3151, *Ne* = 1.1823, *He* = 0.1060, *I* = 0.1589) had the lowest genetic diversity. The mean *Ht*, *Hs*, and *Gst* values were 0.2752, 0.2396, and 0.4926, respectively. The *Nm* value was 0.5150, less than 1, indicating limited gene exchange among the populations. By AMOVA analysis, the genetic difference coefficient (PhiPT value) was found to be 0.490 (*p* < 0.01) and about equal to the *Gst* value. The results demonstrated 49% variance among populations and 51% variance within populations (Table 3).

### 2.3. Population Cluster Analysis

**Figure 1:**
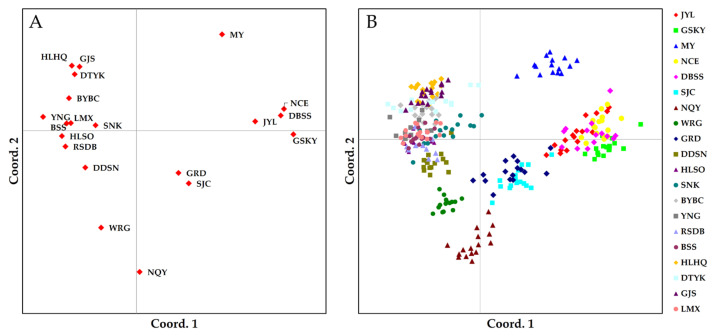
PCoA based on genetic distance was used to visualize the relationship between 20 populations (**A**) and between 300 individuals (**B**).

The computed genetic distance matrix of 20 populations and 300 individuals is provided in Table S1. The genetic distance of 20 populations ranged from 116.788 (*pop* BYBC/RSDB) to 184.004 (*pop* MY/NQY). The resulting distance matrix was visualized using PCoA. The results of the population cluster (Figure 1A) were highly consistent with those of individuals (Figure 1B). The PCoA for 20 populations of *S. medusa* showed that these populations could not be divided into distinct subgroups. However, the population aggregation had certain regional characteristics, such as 11 populations from the western Qilian Mountains and 4 populations from the east. In addition, based on the spatial representation of genetic distance, both populations and individuals in *pop* GRD and *pop* SJC were highly coincident. In contrast, *pop* MY was further separated from other populations.

**Figure 2:**
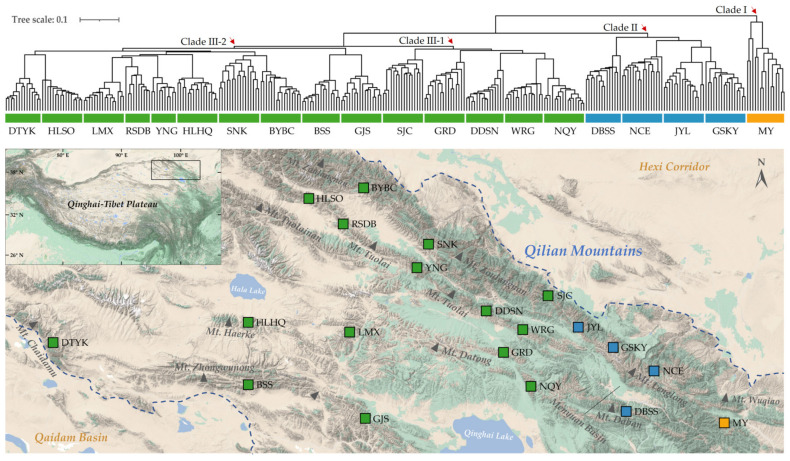
UPGMA dendrogram based on genetic distance values shows the relationship among 300 accessions of *S. medusa*. The orange block denotes Clade I; the blue block denotes Clade II; and the green block denotes Clade III. The area enclosed within the dashed lines in the topographic map represents the sampling sites in the Qilian Mountains.

A dendrogram constructed using the corresponding genetic similarity coefficients obtained from the UPGMA analysis was used to determine the genetic associations in and between the populations of 300 accessions. The generated UPGMA dendrogram categorized the 300 accessions into 3 main phylogenetic clades (Clades I–III; Figure 2). Clade I, represented by *pop* MY, was separated as a distinct outgroup, forming an independent branch. Clade II comprised 4 populations (*pop* GSKY, JYL, NCE, and DBSS) from the alpine area surrounding the Menyuan Basin, while Clade III included 15 populations divided into 2 sister clusters located in the central (Clade III-1: *pop* SJC, NQY, WRG, DDSN, and GRD) and western (Clade III-2: *pop* YNG, HLHQ, LMX, SNK, BYBC, RSDB, HLSO, BSS, DTYK, and GJS) Qilian Mountains.

**Figure 3:**
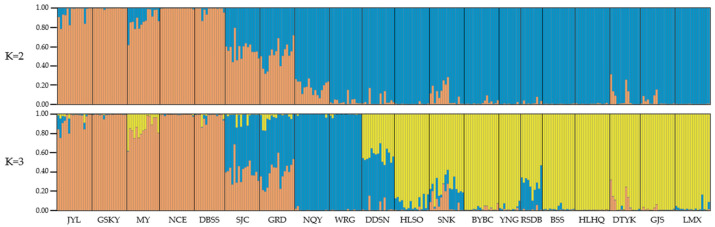
Population structure analysis of 300 *S. medusa* accessions using STRUCTURE packages (K = 2 or 3). The Q value of each color bar describes the probability of membership fractions of each cluster. When the K was 2, there were 2 single-lineage dominant clusters (orange or blue) and the remaining mixed-lineage cluster. When the K was 3, there were 3 single-lineage dominant clusters (orange, blue, or yellow) and the remaining mixed-lineage cluster.

An admixture simulation model was used to assess the clustering of the 300 accessions. Log mean probability and change in log probability (ΔK) were determined using STRUCTURE HARVESTER. A 1–20 K cluster range was evaluated; the output showed a sharp peak with the highest ΔK at K = 2 (Figure S2). Subsequently, a Bayesian bar graph was used to represent the admixture model. Each bar color with ≥0.7 probability of membership fractions (Q value) represented a subgroup in the structural analysis results at K = 2, while accessions with a Q value of <0.7 have mixed ancestry (>1 cluster). Out of 300 accessions, 78 formed Cluster I (orange color, representing 26.0% of the total number of accessions), 190 accessions formed Cluster II (blue color, 63.3%), and the other 32 accessions appeared to have descended from multiple clusters. Cluster I mainly contained accessions sourced from the east of the Qilian Mountains (*pop* JYL, GSKY, MY, NCE, and DBSS). Cluster II members were mostly from the central and western regions of the Qilian Mountains (*pop* NQY, WRG, DDSN, HLSO, SNK, BYBC, YNG, RSDB, BSS, HLHQ, DTYK, GJS, and LMX). For K = 3, of the 300 accessions, 76 appeared in Cluster I (orange color, 25.3%), 34 in Cluster II (blue color, 11.3%), 136 in Cluster III (yellow color, 45.3%), and the remaining accessions were assigned to the mixed-lineage cluster (Figure 3).

### 2.4. Mantel Test

**Figure 4:**
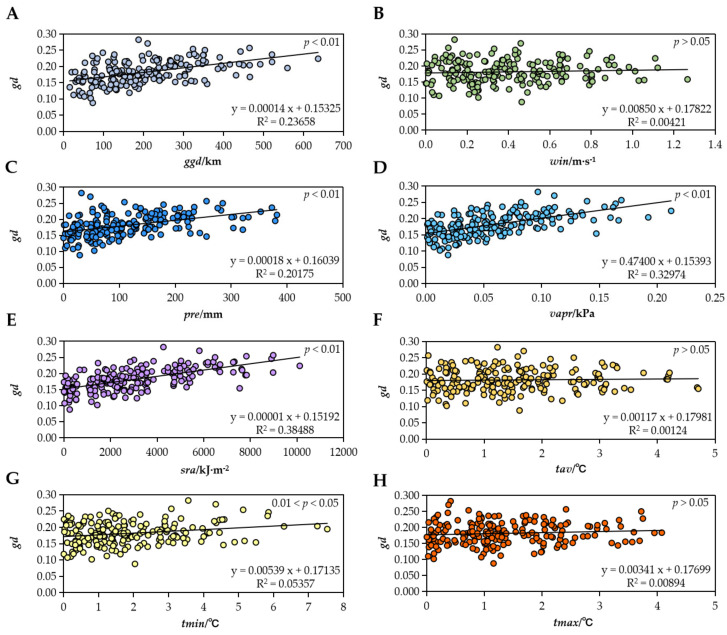
Mantel analysis of geographical distance, climate factor difference, and genetic distance among populations. (**A**) *gd* vs. *ggd*; (**B**) *gd* vs. *win*; (**C**) *gd* vs. *pre*; (**D**) *gd* vs. *vapr*; (**E**) *gd* vs. *sra*; (**F**) *gd* vs. *tav*; (**G**) *gd* vs. *tmin*; (**H**) *gd* vs. *tmax*.

Table S2 shows the *ggd* data of 20 populations, which ranged from 15.176 km (*pop* SNK/YNG) to 636.504 km (*pop* MY/DTYK). Table S3 provides the climatic variable data for the 20 sampling points. The Mantel test results demonstrated a significant or highly significant correlation between *gd* and *ggd* (R^2^ = 0.1942, *p* < 0.01), *gd* and *pre* (R^2^ = 0.2018, *p* < 0.01), *gd* and *vapr* (R^2^ = 0.3297, *p* < 0.01), *gd* and *sra* (R^2^ = 0.3849, *p* < 0.01), and *gd* and *tmin* (R^2^ = 0.0536, 0.01 < *p* < 0.05). However, no correlation was found between *gd* and *win* (R^2^ = 0.0042, *p* > 0.05), *gd* and *tav* (R^2^ = 0.0012, *p* > 0.05), and *gd* and *tmax* (R^2^ = 0.0089, *p* > 0.05) (Figure 4).

## 3. Discussion

### 3.1. Genetic Diversity of S. medusa

The plant population in an isolated “sky island” distribution state usually has low genetic diversity because of the limited gene flow and colonization opportunities of new sites [38]. To date, the genetic diversity and population structure of *S. medusa* have not been reported. In the present study, 14 pairs of SRAP primers were used to analyze the genetic diversity and structure of 300 *S. medusa* accessions from 20 populations. A total of 511 loci were amplified, of which 496 (97.06%) were polymorphic; using this loci information, genetic diversity indices were calculated. Indices such as *Na*, *Ne*, *He*, and *I* exhibited marked variability among sample collections and revealed genetic diversity within and among the sampled populations. The results from a species perspective showed low genetic diversity of *S. medusa* with an *He* of 0.2757, lower than the average *He* of 0.65 for outcrossing plant species and the *He* of 0.55 for short-lived perennials observed in previous genetic studies [39]. From a population-level perspective, the genetic diversity of the *S. medusa* populations located in the western Qilian Mountains (i.e., mountain areas around Qaidam Basin) was significantly lower than that in the central and eastern regions. In particular, *pop* LMX from the west had the lowest genetic diversity, while *pop* SJC from the east–central regions had the highest genetic diversity. The low diversity in the western populations could be linked to low gene flow with central or eastern populations due to geographic or ecological isolation by the existence of valleys in the western Qilian Mountains. Simultaneously, there are no serious geographical barriers between central and eastern native populations, and the distribution of populations was relatively concentrated. Thus, the frequent gene flow among these populations reduced the genetic divergence among populations but enhanced the genetic diversity within them.

### 3.2. Genetic Structure of S. medusa

Different breeding methods determine the genetic variation within a species, such as in plants that are mainly selfing or inbred, where the genetic variation mainly occurs among populations [40]. A study on the breeding system found that selfing was observed in *S. medusa*, although outcrossing was dominant due to the mechanism of herkogamy and dichogamy [21]. *Gst* represents the proportion of interpopulation variation in the total genetic variation of a species and is the most commonly used indicator to measure population genetic differentiation and structure. An increasing *Gst* indicates a greater degree of differentiation between species populations, and a *Gst* of >0.25 suggests significant genetic differentiation [41]. According to the results of Hamrick [42] and Nybom [39], species with a *Gst* of approximately 0.5 exhibit certain selfing, which confirms the existence of selfing in *S. medusa* (*Gst =* 0.4926). Selfing in the absence of pollinators can promote the growth of individual populations to ensure species reproduction. This growth strategy is beneficial to gain a competitive advantage in this niche, as observed in other alpine plants such as *Gentiana lawrencei* var. Farreri, *G. straminea* [43], and *Pedicularis dunniana* [44]. However, the low quality of seeds produced by selfing in the absence of pollinators leads to a decrease in the population growth rate of *S. medusa* [21]. This may explain its small population size.

### 3.3. Population Differentiation and Influencing Factors

To clarify the genetic relationships of the 300 accessions from 20 populations, we used 3 different clustering methods. Using the two-dimensional PCoA distance matrix to visualize the relationship between samples made it difficult to subgroup and analyze them. In contrast, cluster analysis using UPGMA and STRUCTURE showed clear separation patterns among the populations. The results, taken as a whole, showed high genetic differentiation between the regions. Individuals were clustered in 3 major subgroups, i.e., representing the single population sampled from the easternmost Qilian Mountains (*pop* MY), the 4 Menyuan Basin populations (*pop* DBSS, NCE, JYL, and GSKY), and the remaining 11 populations from the central and western Qilian Mountains. In addition, two populations, such as *pop* SJC and JYL, without obvious geographical isolation in the same subgroup, also had greater genetic differentiation. Conversely, populations with geographical isolation had similar genetic backgrounds, for example *pop* MY showed mixed lineages from populations in the central and western Qilian Mountains when K = 3. This confirmed that geographical distance was not the only explanatory factor responsible for the genetic differentiation of *S. medusa* populations. Genetic differentiation may also result from adaptive allele frequency generated by natural selection [45].

We collected data on climate factors in different populations, which showed significant differences in the environment of different habitats. Based on the correlation analysis results, we conclude that differences in moisture conditions such as *pre* and *vapr* (Figure 4C,D) and photothermal conditions such as *sra* and *tmin* (Figure 4E,G) may exacerbate genetic differentiation among *S. medusa* populations. The Qilian Mountains have a typical plateau continental climate. The hydrothermal condition is unique in this area because of the complex terrain. The annual mean temperature is very low (most areas are below 0 °C), but the annual solar radiation is extremely high, exceeding 2800 h. Annual precipitation diminishes from east to west and increases with elevation [46]. The differences in the moisture–photothermal conditions mentioned above may be an important reason for the genetic differentiation of *S. medusa* populations between regions. A study on Qilian juniper (*Sabina przewalskii*) in the semiarid eastern Qaidam Basin found that precipitation, temperature, and growing-season direct solar radiation had potential impacts on its radial growth [47]. Adequate water content is important at various stages of plant development, including the breaking of seed dormancy, seed germination, morphogenesis, and overwintering. In addition, we observed that water was also a key factor in the seed dispersal of *S. medusa*. Although the achenes had a dense pappus adapted to wind propagation, the seedlings were mostly distributed on either side of the water erosion line, showing that water had a certain auxiliary effect on the seed dispersal of *S. medusa*. During the vegetative growth stage, the leaves of *S. medusa* grow as a rosette. At the reproductive stage, *S. medusa* completes the rapid elongation of the aboveground shoot and development of floral organs in a few months (from May to September every year), during which it needs to absorb sufficient solar radiation. However, high temperatures caused by strong solar radiation can reduce pollen viability [48]. *S. medusa* from the western end of the Qilian Mountains is also located in the northern part of the Qaidam Basin, which has an arid/semiarid climate, receiving more solar radiation during the reproductive stage than *S. medusa* growing in the central and eastern Qilian Mountains. Finally, in arctic or alpine environments, the low temperature slows the development of flowers and seeds [15,16,49]. The minimum temperature exerts selective pressure because it determines whether *S. medusa* can survive the coldest month. This may be possible through the development of spectacular pubescence or other thermal insulation systems that have been studied by Tsukaya [15] and Yang [16]. Based on the above, the interaction between factors related to the moisture–photothermal conditions and population differentiation of *S. medusa* deserves further research.

### 3.4. Conservation of S. medusa

In the summer, flowing stones are prone to loosening and collapsing due to rainwater erosion. During the field sampling, we observed many *S. medusa* seeds and seedlings being washed out by flowing water or piled up under flowing rocks. The limited seed dispersal method of this species as well as the decrease in the seed settling rate caused by selfing or inbreeding greatly increases the difficulty of population expansion. Although the Qilian Mountains have the densest distribution of *S. medusa*, frequent human activity and overgrazing have also contributed to its population reduction. We hope that these observations together with the present study’s findings can draw the attention of the International Union for Conservation of Nature (IUCN) to this species, and then the protected areas and a core germplasm resource bank of alpine plants can be established in the future. The present study also suggests that researchers should pay attention to the genetic variation differences caused by different habitat environments when evaluating the genetic diversity of a species.

### 3.5. Limitations of the Study

This study has certain limitations. First, it is difficult to obtain enough *S. medusa* samples from all regions, covering the platform and edge areas of the QTP. We could not avoid estimation errors in the overall genetic diversity assessment. Second, due to the differences in the number and distribution uniformity of amplification sites, using a single molecular marker is often insufficient. Nowadays, genetic diversity analysis methods based on sequencing technology are used in plants, making it possible to obtain more differential site information at the whole-genome level. We look forward to more novel technologies being applied to evaluate the genetic characteristics of *S. medusa* in the future. Third, morphologic and geological contrasts were not taken into account when studying the influencing factors of population differentiation. Despite some of the limitations in the study, the results provide useful outcomes and knowledge that can guide government officials to develop strategies for in situ and ex situ conservation of *S. medusa.*

## 4. Materials and Methods

### 4.1. Sample Information

**Figure 5:**
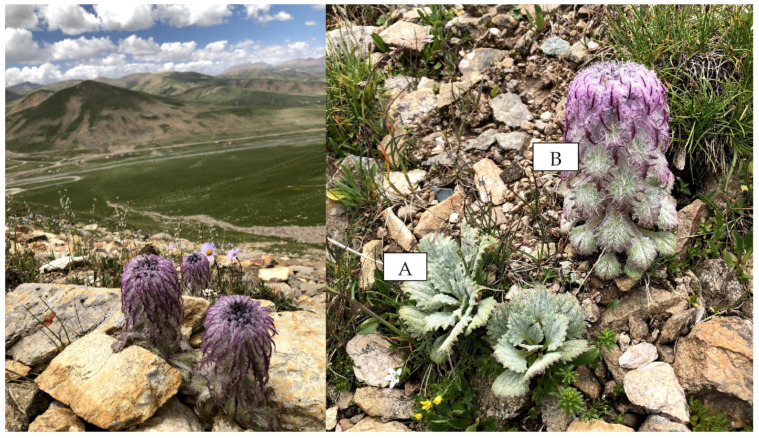
The habitat and plant morphology of *S. medusa*. (**A**) seedling stage; (**B**) reproductive stage.

A total of 300 leaf accessions of *S. medusa* seedlings were collected from 20 natural populations in the Qilian Mountains from east to west. Figure 5 showed the habitat of *S. medusa* and morphology of two growth stages of it. Individuals in each population were randomly sampled at an interval of ≥10 m. We recorded the geographical coordinates and altitude of each sample population using GPS, provided in Table 4. The collected samples were individually placed into non-woven bags with dry silica gel and stored at room temperature. A voucher specimen was deposited in the Qinghai–Tibetan Plateau Museum of Biology, Northwest Institute of Plateau Biology, CAS (voucher specimen #: QPMB 0334412).

### 4.2. Genomic DNA Extraction and SRAP Amplification

The genomic DNA of each individual was manually extracted using a modified CTAB method suitable for medicinal plant [50]. The quantity and quality of the genomic DNA were evaluated using Nanodrop 2000 and 1% agarose gel electrophoresis, respectively. Among 88 pairs, 14 pairs of primer combinations with high polymorphism and good band definition were selected for SRAP amplification. The 20 μL PCR reaction system contained 1.25 U Taq DNA polymerase, 0.3 mM dNTPs, 2 mM 10× buffer (Takara, Beijing, China), 0.4 μM upstream and downstream primers (Sangon, Shanghai, China), 40 ng DNA template, and the rest was filled with ultrapure water. The procedure of SRAP-PCR amplification was based on that used on other Asteraceae species and was appropriately modified [36, 51, 52]: Initial denaturation at 94 °C for 5 min followed by 5 cycles of denaturation at 94 °C for 1 min, annealing at 35 °C for 1 min, and extension at 72 °C for 1 min. Then, for the next 35 cycles, an initial denaturation at 94 °C for 1 min, annealing at 48 °C for 1 min, and extension at 72 °C for 1 min followed by a final extension step at 72 °C for 10 min. The amplified products were stored at 4 °C.

After amplification, the PCR products were separated by 8% polyacrylamide gel electrophoresis (PAGE) followed by dying in 1× Tris-Borate-EDTA buffer (TBE) with ethidium bromide for several seconds. Then, the gel was imaged using the ChemiDoc MP Imaging System (Bio-Rad, Hercules, CA, USA).

### 4.3. Statistical Analysis

Amplified fragments were scored according to the presence (1) or absence (0) of homologous bands; only reproducible bands were considered. Then, band data were transformed into a binary matrix for each primer combination or population [53].

POPGENE 1.32 [54] was used to compare the amplification results of different primer combinations including the size of amplified fragments, the number of loci, and the percentage of polymorphic sites. The *PIC* was calculated as *PIC* = 1 − *p*^2^ − *q*^2^, (*p* was the proportion of bands; *q* was the proportion of no bands) [55]. Genetic indexes such as percentage of polymorphism bands (*PPB*), number of alleles (*Na*), effective number of alleles (*Ne*), Nei’s gene diversity (*He*), and Shannon’s information index (*I*) were calculated to estimate the genetic diversity within different populations of *S. medusa*. Total gene diversity (*Ht*), diversity within a population (*Hs*), and coefficient of genetic differentiation (*Gst*) were calculated as well. The gene flow (*Nm*) was calculated according to the following formula: *Nm* = 0.5(1 − *Gst*)/*Gst*.

The genetic distance (*gd*) matrix of 20 populations was calculated using the distance-based module in GenAlex 6 [56]. Then, molecular analysis of variance (AMOVA) was used to evaluate the contribution of genetic variation among and within populations. Principal coordinate analysis (PCoA) was subsequently performed based on the above results. An unweighted pair group method with arithmetic mean (UPGMA) trees based on populations and individuals was used for SHAN cluster analysis using NTSYS 2.1 [57]. STRUCTURE 2.3.4 [58] was used to analyze the original binary data to calculate the population structure. The length of the Burn-in Period and the amount of MCMC Reps after Burn-in were both set to 10,000. The initial K value was set from 1 to 20, and the number of iterations was set to 10. Then, the running results were uploaded to the online program of STRUCTURE HARVESTER (http://taylor0.biology.ucla.edu/structureHarvester/, accessed on 5 September 2021) to identify the optimal cluster number (ΔK).

In GenAlex, the population coordinate information was input to calculate the tri-matrix of geographic distance (*ggd*). ArcMap 10.6 (ESRI, Redlands, CA, USA) was used to obtain the average annual climate data of 20 sampling points from the WorldClim online database (https://www.worldclim.org/data/worldclim21.html, accessed on 9 February 2021) with a 2.5 arc-minute resolution. According to the needs of the calculation, the above data were sorted into seven climate factors: annual average wind speed (*win*), total annual precipitation (*pre*), annual average water vapor pressure (*vapr*), daily average solar radiation during the growth phase from May to September (*sra*), annual average temperature (*tav*), annual minimum temperature (*tmin*), and annual maximum temperature (*tmax*). The Mantel test revealed the correlation between genetic differentiation and geographical distance, as well as genetic differentiation and the seven climatic factors.

## 5. Conclusions

A low population genetic diversity is reported for the rare monocarpic perennial *S. medusa* in the Qilian Mountains, which can best be explained by its isolated habitats and limited gene flow. In addition, we observed genetic differentiation among populations and a clear west–east differentiation between population groups. Geographic distance and moisture–photothermal conditions may also play key roles in the population differentiation of *S. medusa*.

## Figures and Tables

**Table 1:** Results of SRAP amplification with 14 primer combinations.

Primer ID	Sequence (5’-3’)	Size of Loci (bp)	Number of Loci	Number of Polymorphic Loci	Percentage of Polymorphic Loci (%)	Polymorphic Information Content
ME1/EM3	TGAGTCCAAACCGGATA/	45–1500	27	25	92.59	0.48
	GACTGCGTACGAATTGAC					
ME1/EM4	TGAGTCCAAACCGGATA/	60–1000	35	35	100	0.49
	GACTGCGTACGAATTTGA					
ME2/EM5	TGAGTCCAAACCGGAGC/	90–1500	40	38	95	0.50
	GACTGCGTACGAATTAAC					
ME2/EM9	TGAGTCCAAACCGGAGC/	45–1300	35	33	94.29	0.49
	GACTGCGTACGAATTCGA					
ME3/EM1	TGAGTCCAAACCGGAAT/	70–1600	34	34	100	0.50
	GACTGCGTACGAATTAAT					
ME3/EM3	TGAGTCCAAACCGGAAT/	110–1500	44	44	100	0.50
	GACTGCGTACGAATTGAC					
ME4/EM3	TGAGTCCAAACCGGACC/	65–1400	44	44	100	0.48
	GACTGCGTACGAATTGAC					
ME4/EM4	TGAGTCCAAACCGGACC/	60–1400	34	34	100	0.50
	GACTGCGTACGAATTTGA					
ME4/EM5	TGAGTCCAAACCGGACC/	65–1300	44	44	100	0.49
	GACTGCGTACGAATTAAC					
ME5/EM2	TGAGTCCAAACCGGAAG/	80–1500	32	31	96.88	0.50
	GACTGCGTACGAATTTGC					
ME6/EM9	TGAGTCCAAACCGGTAA/	80–1600	38	36	94.74	0.50
	GACTGCGTACGAATTCGA					
ME7/EM11	TGAGTCCAAACCGGTCC/	80–1500	46	45	97.83	0.50
	GACTGCGTACGAATTCCA					
ME8/EM7	TGAGTCCAAACCGGTGC/	90–1100	32	30	93.75	0.49
	GACTGCGTACGAATTCAA					
ME8/EM9	TGAGTCCAAACCGGTGC/	70–900	26	23	88.46	0.50
	GACTGCGTACGAATTCGA					

**Table 2:** Genetic diversity within 20 natural populations of *S. medusa*

Pop ID	Number of Loci	*PPB* (%)	*Na*	*Ne*	*He*	*I*
JYL	225	44.03	1.4403	1.2555	0.1478	0.2214
GSKY	230	45.01	1.4501	1.2624	0.1534	0.2302
MY	268	52.45	1.5245	1.2685	0.1623	0.2485
NCE	267	52.25	1.5225	1.2882	0.1712	0.2589
DBSS	237	46.38	1.4638	1.2602	0.1536	0.2317
SJC	284	55.58	1.5558	1.3279	0.1913	0.2865
NQY	185	36.20	1.3620	1.2340	0.1322	0.1949
WRG	171	33.46	1.3346	1.1921	0.1129	0.1698
GRD	245	47.95	1.4795	1.2789	0.1630	0.2444
DDSN	201	39.33	1.3933	1.2030	0.1213	0.1846
HLSO	178	34.83	1.3483	1.2071	0.1202	0.1796
SNK	274	53.62	1.5362	1.3155	0.1834	0.2745
BYBC	215	42.07	1.4207	1.2277	0.1339	0.2028
YNG	166	32.49	1.3249	1.1978	0.1145	0.1708
RSDB	205	40.12	1.4012	1.2476	0.1423	0.2116
BSS	176	34.44	1.3444	1.2038	0.1174	0.1754
HLHQ	192	37.57	1.3757	1.2001	0.1196	0.1819
DTYK	164	32.09	1.3209	1.1940	0.1130	0.1688
GJS	199	38.94	1.3894	1.2290	0.1330	0.1989
LMX	161	31.51	1.3151	1.1823	0.1060	0.1589
Species level	511	97.06	1.9706	1.4598	0.2757	0.4237

Note: *PPB*: percentage of polymorphism bands; *Na*: the number of alleles; *Ne*: effective number of alleles; *He*: Nei’s gene diversity; *I*: Shannon’s information index.

**Table 3:** AMOVA analysis of 20 pops of *S. medusa* with SRAP data.

Source	*df*	*SS*	*MS*	Estimated Variance	Percentage (%)	*p*
Among Pops	19	10,753.825	565.991	35.311	49	<0.01
Within Pops	280	10,282.249	36.722	36.722	51	<0.01
Total	299	21,036.073	-	72.033	100	-

**Table 4:** The geographic information of 20 *S. medusa* populations in the Qilian Mountains.

Pop ID	Latitude	Longitude	Altitude (m)	Number of Individuals	Administrative Area
MY	37.0735	102.6701	3805	15	Tianzhu, Gansu Province, China
NCE	37.5328	101.8666	4039	16	Menyuan, Qinghai Province, China
GSKY	37.6805	101.4408	3907	16	Menyuan, Qinghai Province, China
DBSS	37.3376	101.4005	3896	14	Datong, Qinghai Province, China
JYL	37.9108	101.1124	3942	16	Qilian, Qinghai Province, China
SJC	38.0418	100.8139	3710	16	Qilian, Qinghai Province, China
WRG	37.8639	100.5091	3958	16	Qilian, Qinghai Province, China
DDSN	38.0131	100.2412	4138	16	Qilian, Qinghai Province, China
HLSO	39.0415	98.2786	4332	16	Qilian, Qinghai Province, China
RSDB	38.7945	98.7413	4155	15	Qilian, Qinghai Province, China
BYBC	39.0089	98.8191	4444	16	Qilian, Qinghai Province, China
SNK	38.6077	99.4821	4106	16	Qilian, Qinghai Province, China
YNG	38.4745	99.4443	3582	10	Qilian, Qinghai Province, China
GRD	37.6989	100.4176	4005	10	Gangcha, Qinghai Province, China
NQY	37.3821	100.5905	3800	15	Gangcha, Qinghai Province, China
LMX	37.8932	98.8517	4254	16	Tianjun, Qinghai Province, China
GJS	37.1764	98.8781	4095	16	Tianjun, Qinghai Province, China
BSS	37.4790	97.4406	4288	15	Delingha, Qinghai Province, China
HLHQ	37.9416	97.5103	4619	16	Delingha, Qinghai Province, China
DTYK	37.7753	95.5148	4161	14	Chaidan, Qinghai Province, China

## Data Availability

Data is contained within the article and Supplementary Materials.

## Supplementary Materials

The following supporting information can be downloaded at https://www.mdpi.com/article/10.3390/plants12132463/s1, Figure S1: examples of PCR amplification results with various primer combinations; Figure S2: estimation of subgroups in 300 *S. medusa* accessions as revealed by STRUCTURE HARVESTER; Table S1: the tri-matrix of genetic distance (*gd*) of 20 S. *medusa* populations; Table S2: the tri-matrix of geographic distance (*ggd*/km) of 20 *S. medusa* populations; Table S3: the climatic variable data for the 20 sampling points.
