# Supplementary material for: Assessment of Genetic Diversity and Genetic Structure of Saussurea medusa (Asteraceae), a “Sky Island” Plant in the Qinghai–Tibet Plateau, Using SRAP Markers
Source: Plants (Basel). 2023 Jun 27;12(13):2463. doi: 10.3390/plants12132463 (PMC10346629; doi:10.3390/plants12132463)
Supplement: Supplementary file 1 [file plants-12-02463-s001.zip › Figure S1.pdf]

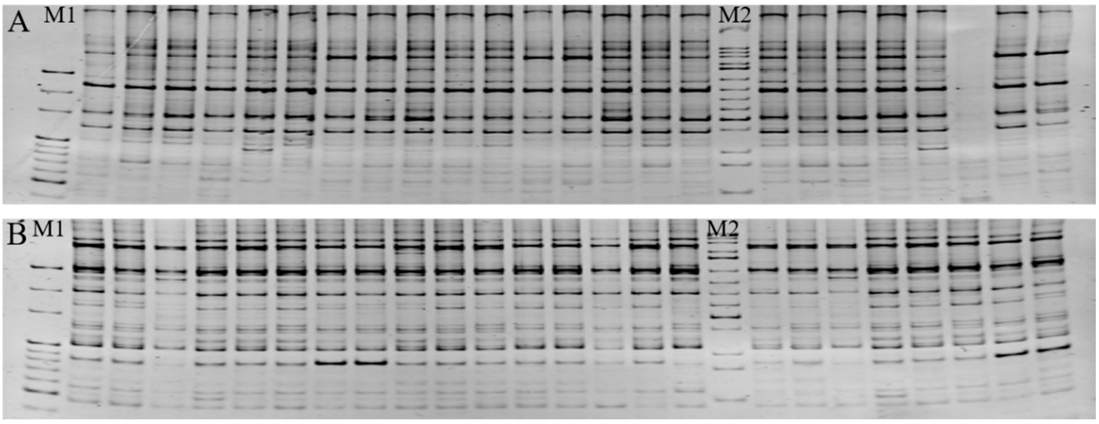

Different primer combinations were used to amplify the same population (*pop* GSKY).

A: ME1/EM3; B: ME1/EM4; M1: 20 bp ladder; M2: 50 bp ladder

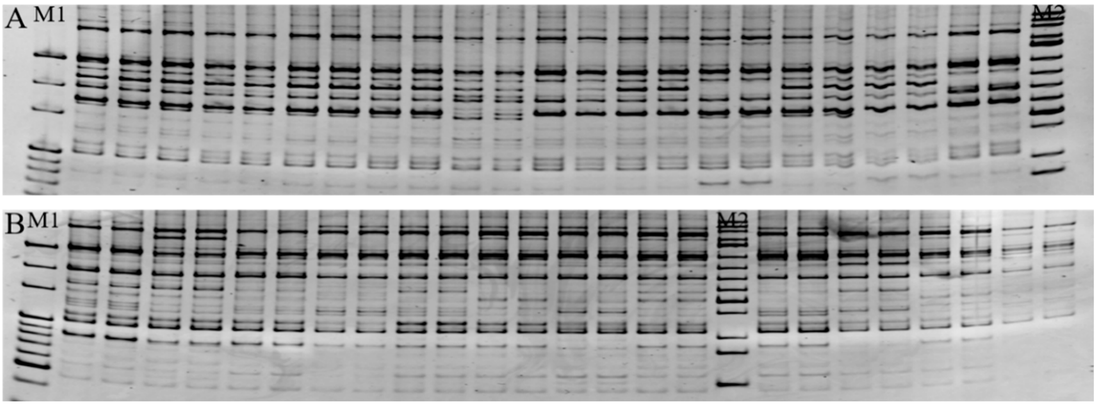

Amplification results of different populations using the same primer combinations (ME1/EM4)

A: *pop* LMX; B: *pop* SJC; M1: 20 bp ladder; M2: 50 bp ladder

Figure S1. Examples of PCR amplification results with various primer combinations.
