# Supplementary material for: Assessment of Genetic Diversity and Genetic Structure of Saussurea medusa (Asteraceae), a “Sky Island” Plant in the Qinghai–Tibet Plateau, Using SRAP Markers
Source: Plants (Basel). 2023 Jun 27;12(13):2463. doi: 10.3390/plants12132463 (PMC10346629; doi:10.3390/plants12132463)
Supplement: Supplementary file 1 [file plants-12-02463-s001.zip › Figure S2.pdf]

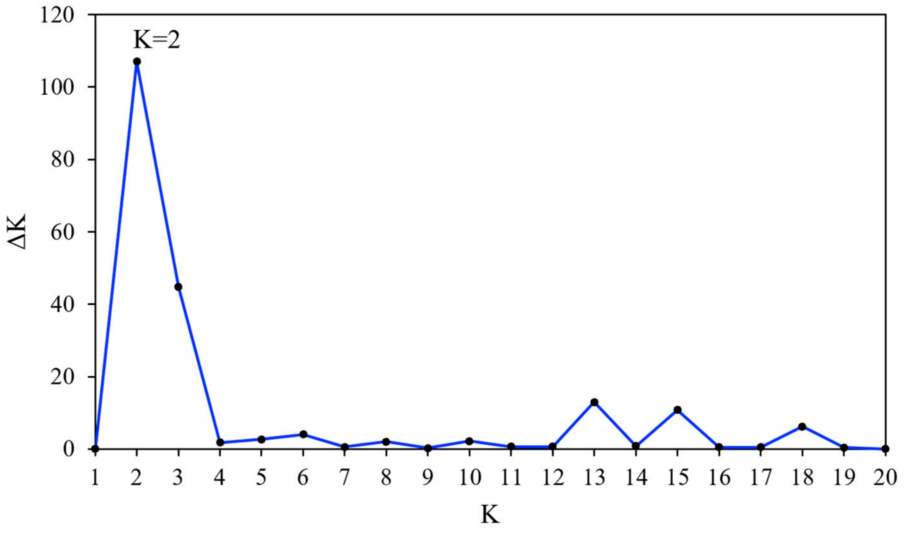

Figure S2. Estimation of subgroups in 300 *S. medusa* accessions as revealed by STRUCTURE HARVESTER. The ΔK generated from K clusters (1–20) showed peaks in optimal subgroups for population structure analysis.
